# Dosimetric Analysis of Proximal Bronchial Tree Subsegments to Assess The Risk of Severe Toxicity After Stereotactic Body Radiation Therapy of Ultra-central Lung Tumors

**DOI:** 10.1016/j.ctro.2023.100707

**Published:** 2023-12-07

**Authors:** Maiwand Ahmadsei, Vinojaa Jegarajah, Riccardo Dal Bello, Sebastian M. Christ, Michael M. Mayinger, Luisa Sabrina Stark, Jonas Willmann, Ivan R. Vogelius, Panagiotis Balermpas, Nicolaus Andratschke, Stephanie Tanadini-Lang, Matthias Guckenberger

**Affiliations:** a Department of Radiation Oncology, University Hospital Zurich, University of Zurich, Zurich, Switzerland; b Center for Proton Therapy, Paul Scherrer Institute, ETH Domain, Villigen, Switzerland; c Department of Oncology, Rigshospitalet, University of Copenhagen, Copenhagen, Denmark

## Abstract

• Stereotactic body radiation therapy (SBRT) for ultra-central lung tumors is associated with high toxicity rates.
• To evaluate differences in radiosensitivity within the proximal bronchial tree (PBT), the PBT was sub-segmented into seven anatomical sections.
• A risk-adapted SBRT regimen of EQD2_10 = 54.4 Gy in 8 or 10 fractions results in excellent local control and low rates of severe toxicity.
• Data from a recent *meta*-analysis, the NORDIC Hilus trial and dosimetric data from this study were combined to create a NTCP model.
• A dose threshold of EQD2_3 = 100 Gy to the PBT or any of its subsegments is expected to result in low rates of severe bronchial toxicity.

## Introduction

Stereotactic body radiotherapy (SBRT) is the treatment of choice for patients with inoperable early stage non-small cell lung cancer (NSCLC) and operable NSCLC, if the risk of surgery is refused by the patient.[1], [2], [3], [4], [5], [6], [7], [8], [9], [10], [11] After SBRT, local control (LC) rates of 80.0 % to 97.0 % after two years are comparable to surgical resection.[12], [13], [14], [15], [16], [17], [18], [19] Furthermore, SBRT is increasingly used in oligometastatic cancer patients.[20], [21], [22], [23], [24], [25], [26], [27], [28], [29], [30] Despite major progress in the field of radiation-oncology, the safety and efficacy of SBRT in ultra-central lung tumors remains controversial.[31], [32].

Almost two decade ago, *Timmerman et al.* described a “No-fly zone” for SBRT in lung tumors located within 2 cm of the proximal bronchial tree (PBT). The authors reported thatSBRT with 60–66 Gy in three fractions to central or perihilar tumors resulted in an 11-fold increased severe toxicity compared to peripheral locations.[33] Later studies confirmed that SBRT to lesions close to the main bronchi resulted in a higher rate of severe toxicity, such as pulmonary hemorrhage and pneumonitis.[31], [34], [35], [36], [37].

Ultra-central lung tumor, a term initially introduced by *Chaudhuri et al.* in 2015, describes tumors directly abutting PBT, trachea or esophagus, but the details of this definition vary in the literature.[38] While some studies have identified ultra-central localization as the planning target volume (PTV) overlapping PBT or esophagus, other studies defined ultra-central localization as PTV overlapping with the PBT, trachea or esophagus - or the gross tumor volume (GTV) overlapping with the PBT, trachea or esophagus.[14], [26], [34], [38] Some studies reported high rates of grade ≥ 3 toxicity and treatment related mortality.[34], [37] In a recent *meta*-analysis by *Chen et al.*[39] analyzing SBRT for ultra-central lung tumors, the median treatment-related grade ≥ 3 toxicity rate was 10 %, with the PBT receiving a EQD2 median dose of 88 Gy. The median treatment-related mortality was 5 %, most commonly from pulmonary hemorrhage. An EQD2 dose of ≥ 108 Gy to the PBT was determined as a high-risk indicator for treatment-related mortality. The prospective phase II *Nordic HILUS trial,* which analyzed the safety and efficacy of SBRT of ultra-central lung tumors within 1 cm of the PBT reported a grade ≥ 3 toxicity rate of 33.8 % and a treatment-related mortality of 15.4 %.[40] Variable definitions of ultra-central localizations, a large heterogeneity of SBRT fractionation schemes and treatment delivery techniques make it difficult to compare published studies - often with small patient numbers and limited follow-up duration.

Ultra-central localization involves the trachea and several bronchial substructures, including carina, main bronchi and lobar bronchi. Interestingly, there is a lack of data on whether the PBT should be considered as one organ at risk (OAR) with one single dose tolerance or whether there is variation in radiation tolerance depending on the PBT anatomical substructures.

Therefore, the aim of this study was to report the outcome of risk-adapted SBRT for ultra-central lung tumor patients and to perform a detailed dosimetric analysis of the PBT and its anatomical substructures and correlate this with observed toxicities after SBRT in patients with ultra-central lung tumors.

## Material and methods

### Patient selection

All patients with ultra-central lung tumors treated and treatment with SBRT at the Department of Radiation Oncology of the University Hospital Zurich (USZ) between 2014 and 2021, were included in this study. An ultra-central lung tumor location was defined as the PTV abutting or overlapping the trachea, PBT and esophagus. All patients presented with either primary inoperable NSCLC, locally recurrent NSCLC or oligometastatic disease (OMD). This project and its design were approved by the Swiss Cantonal Ethics Committee before study initiation (BASEC# 2018–01794).

### Treatment planning and delivery

All patients were treated according to our institutional protocol and underwent three-dimensional (3D) and four-dimensional (4D) computer tomography (CT) simulation to assess breathing motion using a Siemens SOMATOM Definition AS Open (Siemens AG, Germany). For immobilization, all patients were positioned in a vacuum cushion and were imaged head-first-supine. In patients with target lesions in the lower or middle lobes of the lung, abdominal compression was used for reduction of abdominal breathing motion. Organs at risk (OAR) were delineated according to RTOG 0236/ROSEL^5^, the PBT was delineated according to *Kong et al.*[41]. Dose volume constraints (DVC) were applied according to the institutional protocol (Table S6*,* supplementary material). For detailed dosimetric analysis of PBT, we sub-segmented the PBT into 7 anatomical subsegments each two centimeters long to increase the spatial resolution of dose delivered to the PBT. The GTV was defined by fusing FDG-PET/CT and the planning CT using lung window in the ARIA® (Varian Medical Systems, Palo Alto, CA). The GTV was contoured on the end-expiration phase and the end-inspiration phase, the internal target volume (ITV) was defined as the fusion of these two contours. The ITV-to-PTV margin was 5 mm. The RTOG’s conformity index (CI) was defined as the 100 % isodose volume divided by the PTV volume. A detailed description of prescribed doses is shown in Table 2. The treatment was delivered using a TrueBeam^TM^ linear accelerator with daily cone-beam CT based image-guided set-up.

### Data collection and outcome measurement

All patients were identified using the in-house SBRT database. General patient, disease and treatment characteristics were extracted from our hospital information system KISIM^TM^. Radiotherapy (RT) specifications, such as fractionation, single dose, total dose and RT volume were extracted from our treatment planning system Eclipse® (Varian, A Siemens Healthineers Company). Toxicity assessment after treatment was conducted according to Common Terminology Criteria for Adverse Events (CTCAE) Version 5. All grade ≥ 3 toxicities were documented in detail with date of occurrence and therapeutic management. LC, progression-free survival (PFS) and distant control (DC) were assessed using regular follow-up Fluorodeoxyglucose (18F)-Positron emission tomography–computed tomography (FDG-PET/CT) or CT, which were conducted every three months during follow-up. Clinical follow-up was conducted six weeks after the end of treatment and afterwards every 3 months with accompanying imaging.

### Statistical analysis

Overall survival (OS) was measured from the time of completion of SBRT until death or last follow-up. PFS was measured from the time point of completion of SBRT until locoregional relapse, distant disease progression, death, or the last follow-up. LC and DC were measured from the time of treatment completion until disease progression or last follow-up. OS, LC and PFS curves were estimated by using Kaplan-Meier method and compared by log-rank test in MedCalc statistical software. Univariate and multivariate analysis were performed using the Cox proportional hazard model MedCalc statistical software (Version 20.114). Cumulative incidence for competing risks and comparison by Graýs test were estimated with R-Studio statistical software (R-package “cmprsk”). Dosimetric data on target volumes and OARs were extracted from ARIA® and analyzed with R-Studio statistical software (Version 2022.07.1 + 554, R-package “DVHmetrics''). Two-sided p-values of ≤ 0.05 were considered as statistically significant.

A dose response model and a time to toxicity model for grade ≥ 3 adverse events were built using Bayesan inference and compared to the results reported by the Nordic HILUS trial. The dose response model was developed with the aim of defining the upper limit for the complication probability in the case of zero observed events [42]. The model details are provided in the supplementary materials.

## Results

### Patient cohort

A total of 57 patients were included in this study. The median age was 67.7 years (range: 33.0–83.0). The most common primary tumor was primary and loco-regionally recurrent NSCLC (n = 27, 47.4 %), followed by oligometastatic NSCLC (n = 10, 17.5 %), colorectal cancer (n = 4, 7.0 %), head-and-neck cancer (n = 4, 7.0 %), melanoma (n = 3, 5.3 %) and other (n = 9, 15.8 %) A total of 30 patients (53.6 %) had OMD. Furthermore, 22.8 % received a thoracic Type-I re-irradiation[43] before index RT. A detailed summary of the baseline patient- and tumor characteristics is shown in Table 1 *and* Table 2.

**Table 1:** General patient-and tumor characteristics.

Parameter	Results (%)
**Total number of patients**	**n** = **57 patients**
**Age at diagnosis in years, median (range)**	67.7 (33.0–83.0)
**Age over 70 years**	29 (50.9)
**Male gender, n (%)**	42 (73.7)
**Median follow-up time in years (range)**	2.2 (0.6-9.3)
**NSCLC (non-metastatic and loco-regionally recurrent)**	27 (47.4)
o Primary, non-metastatic NSCLC o Adenocarcinoma o Squamous-cell carcinoma	12 (21.1)
	5 (8.8)
	7 (12.3)
o Loco-regionally recurrent NSCLC o Adenocarcinoma o Squamous-cell carcinoma o Large-cell carcinoma	15 (26.3)
	10 (17.5)
	4 (7.0)
	1 (1.8)
**Oligometastatic disease**	30 (52.6)
o NSCLC o Adenocarcinoma o Large-cell carcinoma	10 (17.5)
	9 (15.8)
	1 (1.8)
o SCLC	1 (1.8)
o Colorectal adenocarcinoma	4 (7.0)
o Head-and-Neck cancer	4 (7.0)
o Melanoma	3 (5.3)
o Sarcoma	3 (5.3)
o Other^1^	5 (8.8)
**Patients alive at time of data analysis**	27 (47.4)
**ECOG-PS before index RT, median (range)**	1 (0–2)
**Smoking status**	
o Current	10 (17.5)
o Former	35 (61.4)
o Never	12 (21.1)
**Symptoms at time of radiotherapy**	
o None	30 (52.6)
o Cough	20 (35.0)
o Dyspnea	6 (10.5)
o Hemoptysis	2 (3.5)

1 Includes prostate cancer, mesothelioma, pancreatic cancer and urothelial cancer.

**Table 2:** Detailed patient and treatment characteristics.

**Parameter**	All Patients, n (%)	Primary, non-metastatic NSCLC, n (%)
**Total number**	57 (100)	12 (100.0)
**Alive**	27 (45.5)	3 (33.3)
**RT of Primary tumor**	27 (47.4)	12 (100.0)
**RT of Metastasis**	30 (52.6)	0 (0.0)
**Recurrent disease**	45 (79.0)	0 (0.0)
**Lung function before radiotherapy**		
o FEV1 (l) median, (range) o FEV1 (%) median, (range) o FCV (l) median, (range) o FCV (%) median, (range)	1.9 (0.7-5.0)	1.3 (0.7-1.8)
	75.0 (27.0-117.0)	52.0 (27.0-85.0)
	3.0 (1.5-6.4)	2.2 (1.5-4.1)
	87.0 (47.0-116.0)	72.0 (47.0-104.0)
**COPD** o Stage 1 o Stage 2 o Stage 3 o Stage 4	21 (36.8)	9 (75.0)
	3 (5.3)	0 (0.0)
	8 (14.0)	4 (33.3)
	7 (12.3)	2 (17.0)
	3 (5.3)	3 (25.0)
**PD-L1 status available** o PD-L1 positive	27 (47.4)	4 (33.3)
	12 (21.1)	0 (0.0)
**FDG-PET staging**	55 (96.5)	12 (100.0)
**UICC 8 Stage (Primary, non-metastatic NSCLC)** o Stadium I o Stadium II	9 (15.8)	9 (75.0)
	3 (5.3)	3 (25.0)
**OMD status** o De-novo o Repeat o Induced	30 (52.6)	/
	8 (14.0)	/
	13 (22.8)	/
	9 (15.3)	/
**Prior treatment** o Surgery o Radiotherapy o Type-I re-irradiation o Chemotherapy o Immunotherapy o Targeted therapy	45 (79.0)	0 (0.0)
	29 (50.9)	0 (0.0)
	20 (35.1)	0 (0.0)
	13 (22.8)	0 (0.0)
	22 (38.6)	0 (0.0)
	13 (22.8)	0 (0.0)
	4 (7.0)	0 (0.0)
o Systemic therapy <6 months before index radiotherapy	17 (30.0)	0 (0.0)
**Previous thoracic radiotherapy**	13 (22.8)	0 (0.0)
**Tumor size in cm, (range)** o <3 cm o 3-7 cm o >7 cm	3.9 (1.0-10.5)	4.7 (1.6-8.0)
	16 (26.7)	2 (16.7)
	37 (66.7)	9 (75.0)
	4 (6.7)	1 (8.3)
**Distance of GTV to PBT and main bronchi <10 mm**	55 (95.0)	12 (100)
**GTV size in cm^3^, (range)**	12.5 (0.6-114.94)	25.7 (0.9-88.8)
**PTV size in cm^3^, (range)Endobronchial disease, n (%)**	30.0 (6.0-199.0)	55.9 (7.2-162.0)
	5 (8.8)	2 (16.7)
**PTV location** o Overlap with PBT o Overlap with trachea o Overlap with heart o Overlap with esophagus o Overlap with Aorta o Overlap with pulmonary artery	57 (100.0)	12 (100.0)
	13 (23.0)	3 (25.0)
	14 (25.0)	3 (25.0)
	11 (19.3)	3 (25.0)
	15 (26.3)	4 (33.3)
	44 (77.2)	6 (50.0)
**Treatment characteristics** o Single dose in Gy, median (range) o Fractions, median (range) o Total dose in Gy (enclosing isodose), median (range) o EQD2_10 Gy_ dose in Gy (enclosing isodose), median (range) o Prescription isodose, mode (range) o V100% of PTV in %, median (range) o D0.1cc of PTV in EQD2_10 Gy_ in Gy, median (range)	5 (3-7.5)	
	8 (5–12)	
	45 (30–60)	
	54.4 (33.0 – 88.0) 65% (65-80)	
	96.0% (74.5-99.4%)	
	86.5 (43.1-120.6)	
**Most frequent fractionation scheme** Eight fractions (8fx) o 8 x 6 Gy@65% o 8 x 5 Gy@65% Ten fractions (10fx) o 10 x 5 Gy@80% o 10 x 4.5 Gy@80%	30 (52.6)	
	13 (22.8)	
	13 (22.8)	
	25 (43.9)	
	8 (14.0)	
	6 (10.5)	

### Treatment parameters

**Fig. 1:**
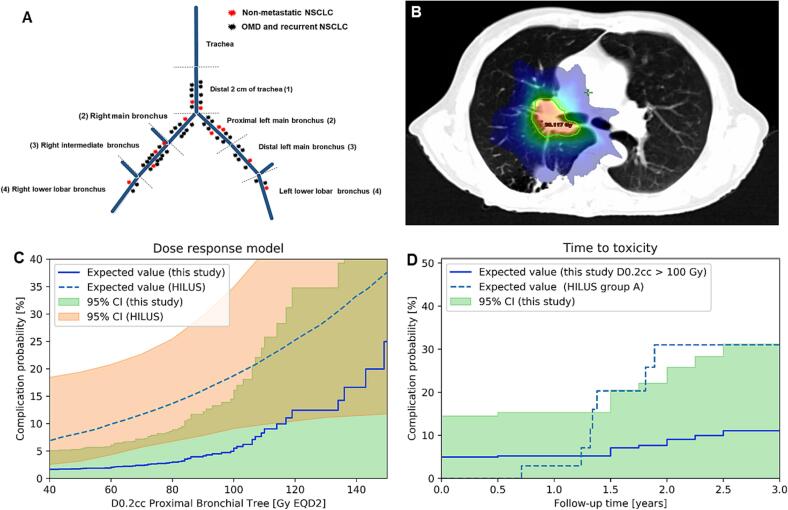
**A-C: Location of treated lesions overlapping the proximal bronchial tree (A), representative example (B), dose**–**response model for grade** ≥ **3 stenosis, hemorrhage or fistula based on dose to the proximal bronchial tree compared to the NTCP model derived from the full cohort of the HILUS trial (C), model including follow-up time for patients receiving more than 100 Gy compared to Group A of HILUS trial (D)**.

The median PTV size for all patients was 30.0 (6.0–199.0) cm^3^. Twenty-seven (47.4 %) of the irradiated lesions were primary lung tumors and 30 (52.6 %) lymph node metastases. A detailed overview of tumor localization is shown in Fig. 1*a and* Table 2*.* The most commonly used fractionations were 8 x 6 Gy@65 % (22.8 %), 8 x 5 Gy@65 % (22.8 %), 10 x 5 Gy@80 % (14.0 %) and 10 x 4.5 Gy @80 % (10.5 %). A detailed overview of dose delivered to OAR are shown in Table 4*.* In order to fulfill the dose constraints for OAR, a compromise in PTV dose coverage was accepted for 9 patients (15.8 %) as shown in Table S7 *(*supplementary material*)*.

### Control rates, overall survival and progression-free survival

**Fig. 2:**
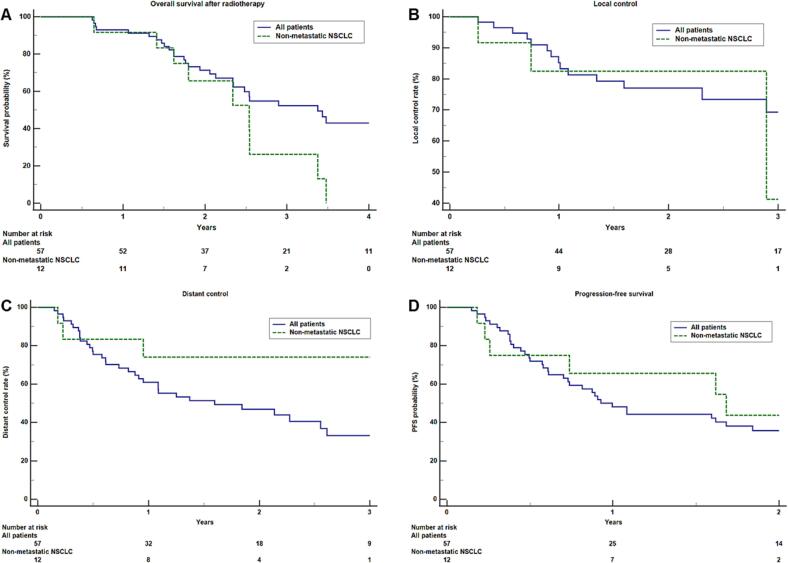
A-D: (A) Kaplan-Meier plot of overall survival, (B) local control rate for all patients and non-metastatic NSCLC, (C) distant control rate and (D) progression-free survival with the number of patients at risk is presented as well.

The median follow-up time was 2.2 (0.6–9.3) years. At the time of analysis, 30 patients (52.6 %) were dead and 39 patients showed disease progression during the follow-up. The median OS for all patients was 3.4 (0.6–9.3) years, the median OS for primary, non-metastatic NSCLC was 2.5 (0.7–3.5) years. The 1- and 2-years LC for all patients were 85.2 % and 77.1 %, respectively. The 2-years LC for primary, non-metastatic NSCLC was 83.0 % and 75.5 % for OMD.The median PFS for all patients was 1.0 (0.2–5.7) year as shown in Fig. 2. A detailed overview of LC, DC, PFS rates and treatment after index RT is shown in Table 3. Of n = 9 patients with a compromise in PTV dose coverage, only one patient developed local failure; compromises to PTV coverage were not associated with worse LC in the Cox regression uni-and multivariate analysis, the summary for uni- and multivariate analysis is shown in Table S5 *(*supplementary material*).*

**Table 3:** Local- and distant control rates, overall survival, progression-free survival and treatment-related toxicity.

	**All Patients**	**Primary, non-metastatic NSCLC**
Median survival from time of radiotherapy years, (range) o 2-year survival (%)	3.4 (0.6-9.3)	2.5 (0.7-3.5)
	71.3	66.0
Median local control in years, (range) o 1-year local control rate (%) o 2-year local control rate (%)	*Not reached*	2.9 (0.3-3.4)
	85.2	83.0
	77.1	83.0
Median distant control in years, (range) o 1-year distant control rate (%) o 2-year distant control rate (%)	1.6 (0.2-5.7)	*Not reached*
	60.0	74.1
	47.0	74.1
Median PFS in years, (range)	1.0 (0.2-5.7)	1.7 (0.2-5.2)
Treatment after index radiotherapy o Surgery o Radiotherapy o Chemotherapy o Immunotherapy o Targeted therapy	34 (60.0)	1 (8.3)
	5 (8.3)	0 (0.0)
	20 (36.7)	0 (0.0)
	18 (33.3)	1 (8.3)
	14 (25.0)	1 (8.3)
	6 (11.7)	0 (0.0)
Systemic therapy <6 months after index radiotherapy	22 (39.0)	1 (8.3)
**Treatment-related toxicity**		
*Type of toxicity*		
Radiation pneumonitis		
o Early Grade 3 o Early Grade 4 o Early Grade 5 o Late Grade 3 o Late Grade 4 o Late Grade 5 Bronchial stenosis o Early Grade 3-5 o Late Grade 3-5 Bronchopulmonary hemorrhage o Early Grade 3-5 o Late Grade 3-5 Fistula formation o Early Grade 3-5 o Late Grade 3-5 Esophagitis o Early Grade 3-5 o Late Grade 3-5	0	
	0	
	0	
	1 (1.7%)	
	1 (1.7%)	
	0	
	0	
	0	
	0	
	0	
	0	
	0	

	0	
	0	

### Toxicity

After completion of SBRT, two patients (3.5 %) experienced late grade ≥ 3 treatment-related toxicities. No grade ≥ 3 esophagitis, fistula, stenosis or bronchopulmonary hemorrhage was observed in this study. All grade ≥ 3 treatment-related toxicities are shown in Table 3. In the absence of SBRT-related grade ≥ 3 toxicity to the PBT, we did not observe any association with dose delivered to a specific PBT subsegment. In this cohort we observed the highest doses (up to EQD2_3 171.5 Gy) delivered to PBT mainly in the distal 2 cm of trachea, right main bronchus and proximal left main bronchus.

We therefore added PBT tolerance doses from the systematic review by *Chen et al*.[39], as well as NTCP models from the *Nordic HILUS trial*[40] to validate those based on our dosimetric results and toxicity rates. A detailed overview of dose delivered to PBT subsegments are shown in Table 4 *and* Table S8 *(*supplementary material*)*.

**Table 4:** Detailed location of lesions, size of PTV overlap with PBT and doses in EQD2 to thoracic OARs.

**Structure**	**Number of lesions (%)**	**Median PTV overlap in cm^3^ (range)**	
Total PTB	57 (100.0)	4.4 (0.5–17.2)	
Total right bronchus	33 (58.0)	3.0 (0.1–15.3)	
Total left bronchus	24 (42.1)	2.7 (0.1–15.0)	
Segment 1 (Distal 2 cm of trachea)	21 (37.0)	1.5 (0.1–4.24)	
Segment 2R (Right main bronchus)	29 (51.0)	1.9 (0.1–9.4)	
Segment 2L (Proximal left main bronchus)	21 (37.0)	1.4 (0.1–5.9)	
Segment 3R (Right intermediate bronchus)	22 (39.0)	2.2 (0.1–8.0)	
Segment 3L (Distal left main bronchus)	18 (32.0)	1.2 (0.1–8.4)	
Segment 4R (Right lower lobar bronchus)	12 (21.1)	0.7 (0.2–3.7)	
Segment 4L (Left lower lobar bronchus)	4 (7.0)	0.5 (0.3–0.7)	
**Structure**	**Dose measured (EQD2, α/β = 3 Gy)**	**Median (range)**	**Number of patients receiving > EQD2_3 = 100 Gy, n (%)**
Trachea	D_0.2cc_	14.6 (0.3–115.2)	1 (1.8)
Proximal bronchial tree	D_0.2cc_	84.2 (44.0–159.3)	18 (31.6)
PBT sub-structures overlapping with PTV o Distal 2 cm of trachea o Right main bronchus o Proximal left main bronchus o Right intermediate bronchus o Distal left main bronchus o Right lower lobar bronchus o Left lower lobar bronchus			
	D_0.2cc_	11.3 (0.2–140.7)	3 (5.3)
	D_0.2cc_	13.3 (0.1–159.2)	3 (5.3)
	D_0.2cc_	19.0 (0.1–156.4)	5 (8.8)
	D_0.2cc_	7.2 (0.1–133.6)	3 (5.3)
	D_0.2cc_	5.4 (0.1–106.5)	3 (5.3)
	D_0.2cc_	9.9 (0.1–126.8)	2 (3.5)
	D_0.2cc_	4.7 (0.1–133.0)	1 (1.8)
Heart	D_max_ D_1.0cc_	19.4 (0.1–125.6) 12.0 (0.01–109.0)	2 (3.5)
Esophagus	D_1.0cc_	12.1 (3.1–76.7)	0 (0.0)

The dose–response model reporting the upper limits for the complication probability and its dependency on follow-up time for grade ≥ 3 esophagitis, fistula, stenosis or bronchopulmonary hemorrhage are shown in Fig. 1*c-d*. The expected risk for the grade ≥ 3 toxicity of the *Nordic HILUS trial*[40] is within the CI of the current study for doses < 20 Gy and > 106 Gy, while the confidence interval (CI) of the two models present agreement over the entire dose range. Doses of EQD2_3 = 100 Gy or lower to any location of the PBT (D0.2 cc) resulted in (Complication probability) CP limited to 0.0 %-14.5 % (95 % CI), which should be compared to 9 %-35 % (95 % CI) reported by the *Nordic HILUS trial*[40]. For this specific dose level the analysis of the follow-up time was also performed. At the median follow-up time for this study CP was limited to 0 %-25.8 % (95 % CI), compared to 4.0 %, 20.0 % and 35.0 % reported by the *Nordic HILUS trial*[40] for tumor location > 10 mm, 6–10 mm and 0–5 mm from the main bronchus, respectively. The prediction of toxicity for longer follow-up times and higher dose levels was performed and reported in Fig. 1*d*, but the prediction power was limited due to the limited number of patients and no grade ≥ 3 toxicity events associated with SBRT of ultra-central lung tumors.

## Discussion

SBRT is a guideline-recommended treatment option for patients with inoperable early stage lung cancer and pulmonary oligometastases. While peripherally located lesions can be treated safely and result in excellent LC after SBRT, treatment of ultra-central lung tumors remains controversial due to high rates of severe SBRT-related toxicity reported.[44] Furthermore, it remains unknown whether the PBT should be considered as a single OAR or specified into anatomical subsegments when reported tolerance doses in the literature.

This retrospective, single-center study conducted a detailed dosimetric analysis of PBT substructures to assess the risk of severe toxicity after SBRT for ultra-central lung tumors and demonstrated favorable safety and efficacy of a SBRT regimen with a median dose of 45.0 (30.0–60.0) Gy in 8 or 10 fractions. At a median follow-up of 2.2 years, 2-years OS was 71.3 %, 1- and 2-year LC were 85.2 % and 77.1 %, respectively. Most importantly, no patient showed any grade ≥ 3 treatment-related toxicities classically associated with SBRT of ultra-central lung tumors, such as bronchial stenosis, bronchopulmonary hemorrhage or fistula formation. A detailed dosimetric analysis of PBT and its substructures, which was conducted to increase the anatomical resolution of dose delivered to the PBT and identify more vulnerable regions within the PBT, showed no association between delivered dose and occurrence of grade ≥ 3 treatment-related toxicities in the absence of events. Therefore, the current study cannot conclude on putative differences in radiation tolerance between the PBT anatomical subsections. The dose–response model including as prior the tolerance doses from recent literature[39] predicted a toxicity limited to 0.0 %-14.5 % (95 % CI) when delivering EQD2_3 = 100 Gy to any location of the PBT. The CI derived in the current study were in agreement with the CI reported by the *Nordic HILUS trial*[40]. It should be noted that in absence of observed toxicity, a comprehensive NTCP model cannot be developed and this was not the aim of the current study. On the other hand, a high number of retrospectively analyzed patients without toxicity correspond to a limited complication probability. Therefore, the Bayesian model presented in the current study quantitatively reports the upper limit for the complication probability as a function of applied dose and follow-up time.

In a recent *meta*-analysis of SBRT for ultra-central lung tumors by *Chen et al.*[39], the median rate of grade ≥ 3 toxicities was 10 %, while the median treatment-related mortality rate was 5 %. Endobronchial disease, use of antiplatelets/anticoagulants, concurrent use of bevacizumab, a recent biopsy and a maximum dose of EQD2_3 = 100 Gy to the PBT were reported as high-risk factors of severe toxicity. The median PTV size of 44.0–104.0 cm^3^ resulting in larger PBT-volume irradiated reported by *Chen et al.*[39], was larger in comparison to the median PTV size of 30.0 (6.0–199.0) cm^3^ in this study, thereby possibly contributing to the finding of an absence of any severe SBRT-related toxicity in the present study. This hypothesis of a relevant volume factor is further supported by the fact that other recent studies reporting higher grade ≥ 3 rates of up to 24.0 % included similarly larger tumor volumes with median PTV sizes two to three times larger compared to median PTV of the present study.[12], [14], [37].

Additionally, the afore mentioned studies, reporting higher treatment-related toxicity rates, not only included larger tumor volumes but also a higher dose prescription of 60.0 Gy in 8 or 12 fractions compared to the present SBRT regimen of 45.0 Gy in 8 or 10 fractions, thereby highlighting the crucial role of careful dose selection. Additional factors leading to high rates of toxicity, such as the presence of interstitial lung disease (ILD) were not present in the studied cohort.[37].

The prospective phase II landmark *Nordic HILUS*[40] trial evaluated the efficacy and safety of SBRT of ultra-central lung tumors 1 cm from the PBT with 56 Gy in 8 fractions prescribed to 67 % isodose line. In this study, the authors reported a high rate of treatment-related mortality of 15.4 % for patients with lesions residing within 10 mm of the trachea or main bronchi. The SBRT regimen of 45 Gy in 8 or 10 fractions in this study resulted in significantly lower rates of severe SBRT-related toxicity while achieving acceptable LC despite the fact 95.0 % of the treated lesions were within 10 mm of PBT, trachea or main bronchi. Our inverse SBRT planning approach furthermore allowed dose escalation strictly limited to the ITV of up to 120.0 %-150.0 % of the prescription dose. To fulfill dose constraints of PBT, PTV-coverage was compromised in 16 % of the patients; however, compromised PTV coverage did not result in lower LC rates in this study. Despite overall slightly lower LC rates in our cohort in cross-study comparison, our results are in the range of previously reported studies.[15] Since our OS rates are in line with previously reported results[45] and the fact that distant disease progression remains the driving factor in limiting survival, a risk-adapted SBRT regimen of 45 Gy in 8 or 10 fractions appears a favorable compromise between safety and efficacy.

The dose response and the time to toxicity models of the current study were based on Bayesian inference assuming a prior distribution derived from previously reported data. This approach allowed not only to compare the toxicity rates to previous studies at fixed dose levels, but also to update the posterior and compute the expected value and confidence interval for the NTCP of grade ≥ 3 treatment-related toxicities such as stenosis, bronchopulmonary hemorrhage or fistula formation. The absence of toxicities at given dose levels in the current cohort allowed to define the likelihood distribution of such events. We report an agreement between the CI of the current study and the HILUS trial (Fig. 1*C*). The higher rates of toxicity in the Nordic Hilus trial might be explained by larger PTV volumes, as well as the fact that the authors used PTV margins of up to 15 mm and compared to the studied cohort a more heterogeneous isodose line prescription of 65 %, thereby increased the risk of 150 % dose hot-spots within the PBT. Future studies are needed to analyze the potential effects of dose hot-spots and their location in the PBT.[46] Due to the absence of relevant toxicity, we conclude that at the dose levels used in the current study a difference of tolerance within the PBT does not appear, yet the application of an escalated dose might reveal differences in tolerance within the PBT.

Some limitations apply to the current study due to its retrospective nature. Furthermore, different SBRT fractionation scheme were used and the patient collective included primary and recurrent NSCLC and also pulmonary oligometastases. Strengths of this study include a rigorous, standardized follow-up protocol including a PET-CT imaging every 3 months, a consistent definition of ultra-central lung tumor patients and detailed dosimetric analysis of the PBT and its substructures.

In conclusion, risk-adapted SBRT Gy in 8 or 10 fractions for ultra-central lung tumors results in a favorable therapeutic ratio of high local tumor control without serious toxicities. Our exploratory dose–response analysis suggests a dose tolerance of EQD2_3 = 100 Gy to the PBT without differences between the PBT anatomical subsegments. Therefore, we propose to consider the PBT as a one single OAR with one single dose tolerance.

## Declaration of competing interest

The authors declare that they have no known competing financial interests or personal relationships that could have appeared to influence the work reported in this paper.

## References

[1] Guckenberger M., Andratschke N., Dieckmann K., Hoogeman M.S., Hoyer M., Hurkmans C. (2017). ESTRO ACROP consensus guideline on implementation and practice of stereotactic body radiotherapy for peripherally located early stage non-small cell lung cancer. Radiother Oncol J Eur Soc Ther Radiol Oncol.

[2] Chang J.Y., Mehran R.J., Feng L., Verma V., Liao Z., Welsh J.W. (2021). Stereotactic ablative radiotherapy for operable stage I non-small-cell lung cancer (revised STARS): long-term results of a single-arm, prospective trial with prespecified comparison to surgery. Lancet Oncol.

[3] Khorfan R., Kruser T.J., Coughlin J.M., Bharat A., Bilimoria K.Y., Odell D.D. (2020). Survival of Primary Stereotactic Body Radiation Therapy Compared With Surgery for Operable Stage I/II Non-small Cell Lung Cancer. Ann Thorac Surg.

[4] Onishi H., Shirato H., Nagata Y., Hiraoka M., Fujino M., Gomi K. (2011). Stereotactic body radiotherapy (SBRT) for operable stage I non-small-cell lung cancer: can SBRT be comparable to surgery?. Int J Radiat Oncol Biol Phys.

[5] Louie A.V., van Werkhoven E., Chen H., Smit E.F., Paul M.A., Widder J. (2015). Patient reported outcomes following stereotactic ablative radiotherapy or surgery for stage IA non-small-cell lung cancer: Results from the ROSEL multicenter randomized trial. Radiother Oncol J Eur Soc Ther Radiol Oncol.

[6] Yu J.B., Soulos P.R., Cramer L.D., Decker R.H., Kim A.W., Gross C.P. (2015). Comparative effectiveness of surgery and radiosurgery for stage I non-small cell lung cancer. Cancer.

[7] Tandberg D.J., Tong B.C., Ackerson B.G., Kelsey C.R. (2018). Surgery versus stereotactic body radiation therapy for stage I non-small cell lung cancer: A comprehensive review. Cancer.

[8] Puri V., Crabtree T.D., Kymes S., Gregory M., Bell J., Bradley J.D. (2012). A comparison of surgical intervention and stereotactic body radiation therapy for stage I lung cancer in high-risk patients: a decision analysis. J Thorac Cardiovasc Surg.

[9] Zheng X., Schipper M., Kidwell K., Lin J., Reddy R., Ren Y. (2014). Survival outcome after stereotactic body radiation therapy and surgery for stage I non-small cell lung cancer: a meta-analysis. Int J Radiat Oncol Biol Phys.

[10] *Ann Surg Oncol*. 2015;22(1):316-323. doi:10.1245/s10434-014-3860-x. 10.1245/s10434-014-3860-x 24962941

[11] Grills I.S., Mangona V.S., Welsh R., Chmielewski G., McInerney E., Martin S. (2010). Outcomes after stereotactic lung radiotherapy or wedge resection for stage I non-small-cell lung cancer. J Clin Oncol off J Am Soc Clin Oncol.

[12] Mihai A.M., Armstrong P.J., Hickey D., Milano M.T., Dunne M., Healy K. (2021). Late Toxicity and Long-Term Local Control in Patients With Ultra-Central Lung Tumours Treated by Intensity-Modulated Radiotherapy-Based Stereotactic Ablative Body Radiotherapy With Homogenous Dose Prescription. Clin Oncol R Coll Radiol G b.

[13] Wang B., Dong Y., Yu X., Li F., Wang J., Chen H. (2022). Safety and Efficacy of Stereotactic Ablative Radiotherapy for Ultra-Central Lung Cancer. Front Oncol.

[14] Lodeweges J.E., van Rossum P.S.N., Bartels M.M.T.J., van Lindert A.S.R., Pomp J., Peters M. (2021). Ultra-central lung tumors: safety and efficacy of protracted stereotactic body radiotherapy. Acta Oncol Stockh Swed.

[15] Rim C.H., Shin I.S., Yoon W.S., Park S. (2020). Dose-response relationship of stereotactic body radiotherapy for ultracentral tumor and comparison of efficacy with central tumor: a meta-analysis. Transl Lung Cancer Res.

[16] Zhao Y., Khawandanh E., Thomas S., Zhang S., Dunne E.M., Liu M. (2020). Outcomes of stereotactic body radiotherapy 60 Gy in 8 fractions when prioritizing organs at risk for central and ultracentral lung tumors. Radiat Oncol Lond Engl.

[17] Cooke R., Camilleri P., Chu K.-Y., O'Cathail S.M., Robinson M., Van Den Heuvel F. (2020). Stereotactic body radiotherapy for moderately central and ultra-central oligometastatic disease: Initial outcomes. Tech Innov Patient Support Radiat Oncol.

[18] Yang D., Cui J., Zhao J., You J., Yu R., Yu H. (2020). Stereotactic ablative radiotherapy of 60 Gy in eight fractions is safe for ultracentral non-small cell lung cancer. Thorac Cancer.

[19] Klement R.J., Sonke J.-J., Allgäuer M., Andratschke N., Appold S., Belderbos J. (2020). Correlating Dose Variables with Local Tumor Control in Stereotactic Body Radiation Therapy for Early-Stage Non-Small Cell Lung Cancer: A Modeling Study on 1500 Individual Treatments. Int J Radiat Oncol Biol Phys.

[20] Klement R.J., Hoerner-Rieber J., Adebahr S., Andratschke N., Blanck O., Boda-Heggemann J. (2018). Stereotactic body radiotherapy (SBRT) for multiple pulmonary oligometastases: Analysis of number and timing of repeat SBRT as impact factors on treatment safety and efficacy. Radiother Oncol J Eur Soc Ther Radiol Oncol.

[21] D.A. Palma Department of Radiation Oncology, London Health Sciences Centre, London, Ontario, Canada. A.V. Louie Department of Radiation Oncology, London Health Sciences Centre, London, Ontario, Canada. G.B. Rodrigues Department of Radiation Oncology, London Health Sciences Centre, London, Ontario, Canada. New Strategies in Stereotactic Radiotherapy for Oligometastases Clin Cancer Res Off J Am Assoc Cancer Res. 21 23 2015 5198 5204. 10.1158/1078-0432.CCR-15-0822 26626571

[22] Palma D.A., Olson R., Harrow S., Gaede S., Louie A.V., Haasbeek C. (2019). Stereotactic ablative radiotherapy versus standard of care palliative treatment in patients with oligometastatic cancers (SABR-COMET): a randomised, phase 2, open-label trial. Lancet Lond Engl.

[23] Gulstene S., Ruwanpura T., Palma D., Joseph N. (2022). Stereotactic Ablative Radiotherapy in the Treatment of Early-Stage Lung Cancer – A Done Deal?. Clin Oncol.

[24] Gomez D.R., Blumenschein G.R., Lee J.J., Hernandez M., Ye R., Camidge D.R. (2016). Local consolidative therapy versus maintenance therapy or observation for patients with oligometastatic non-small-cell lung cancer without progression after first-line systemic therapy: a multicentre, randomised, controlled, phase 2 study. Lancet Oncol.

[25] Iyengar P., Wardak Z., Gerber D.E., Tumati V., Ahn C., Hughes R.S. (2018). Consolidative Radiotherapy for Limited Metastatic Non-Small-Cell Lung Cancer: A Phase 2 Randomized Clinical Trial. JAMA Oncol.

[26] Mihai A., Mu Y., Armstrong J. (2017). Patients with colorectal lung oligometastases (L-OMD) treated by dose adapted SABR at diagnosis of oligometastatic disease have better outcomes than patients previously treated for their metastatic disease. J Radiosurgery SBRT.

[27] Sutera P., Clump D.A., Kalash R., D'Ambrosio D., Mihai A., Wang H. (2019). Initial Results of a Multicenter Phase 2 Trial of Stereotactic Ablative Radiation Therapy for Oligometastatic Cancer. Int J Radiat Oncol Biol Phys.

[28] Ost P., Jereczek-Fossa B.A., Van As N., Zilli T., Tree A., Henderson D. (2016). Pattern of Progression after Stereotactic Body Radiotherapy for Oligometastatic Prostate Cancer Nodal Recurrences. Clin Oncol R Coll Radiol G b.

[29] Ost P., Reynders D., Decaestecker K., Fonteyne V., Lumen N., De Bruycker A. (2018). Surveillance or Metastasis-Directed Therapy for Oligometastatic Prostate Cancer Recurrence: A Prospective, Randomized, Multicenter Phase II Trial. J Clin Oncol off J Am Soc Clin Oncol.

[30] Siva S., Senan S., Ball D. (2015). Ablative therapies for lung metastases: a need to acknowledge the efficacy and toxicity of stereotactic ablative body radiotherapy. Ann Oncol off J Eur Soc Med Oncol.

[31] Palma D., Daly M., Urbanic J., Giuliani M. (2019). Stereotactic Radiation for Ultra-Central Lung Tumors: Good Idea, or Ultra-Risky?. Int J Radiat Oncol Biol Phys.

[32] Stam B., Kwint M., Guckenberger M., Mantel F., Hope A., Giuliani M. (2019). Subgroup Survival Analysis in Stage I-II NSCLC Patients With a Central Tumor Partly Treated With Risk-Adapted SBRT. Int J Radiat Oncol.

[33] Timmerman R., McGarry R., Yiannoutsos C., Papiez L., Tudor K., DeLuca J. (2006). Excessive toxicity when treating central tumors in a phase II study of stereotactic body radiation therapy for medically inoperable early-stage lung cancer. J Clin Oncol off J Am Soc Clin Oncol.

[34] Bezjak A., Paulus R., Gaspar L.E., Timmerman R.D., Straube W.L., Ryan W.F. (2019). Safety and Efficacy of a Five-Fraction Stereotactic Body Radiotherapy Schedule for Centrally Located Non-Small-Cell Lung Cancer: NRG Oncology/RTOG 0813 Trial. J Clin Oncol off J Am Soc Clin Oncol.

[35] Lindberg K., Bergström P., Brustugun O.T., Engelholm S., Grozman V., Hoyer M. (2017). OA24.05 The Nordic HILUS-Trial - First Report of a Phase II Trial of SBRT of Centrally Located Lung Tumors. J Thorac Oncol.

[36] Roesch J., Panje C., Sterzing F., Mantel F., Nestle U., Andratschke N. (2016). SBRT for centrally localized NSCLC - What is too central?. Radiat Oncol Lond Engl.

[37] Tekatli H., Haasbeek N., Dahele M., De Haan P., Verbakel W., Bongers E. (2016). Outcomes of Hypofractionated High-Dose Radiotherapy in Poor-Risk Patients with “Ultracentral” Non-Small Cell Lung Cancer. J Thorac Oncol off Publ Int Assoc Study Lung Cancer.

[38] Chaudhuri A.A., Tang C., Binkley M.S., Jin M., Wynne J.F., von Eyben R. (2015). Stereotactic ablative radiotherapy (SABR) for treatment of central and ultra-central lung tumors. Lung Cancer Amst Neth.

[39] Chen H., Laba J.M., Zayed S., Boldt R.G., Palma D.A., Louie A.V. (2019). Safety and Effectiveness of Stereotactic Ablative Radiotherapy for Ultra-Central Lung Lesions: A Systematic Review. J Thorac Oncol.

[40] Lindberg K., Grozman V., Karlsson K., Lindberg S., Lax I., Wersäll P. (2021). The HILUS-Trial-a Prospective Nordic Multicenter Phase 2 Study of Ultracentral Lung Tumors Treated With Stereotactic Body Radiotherapy. J Thorac Oncol off Publ Int Assoc Study Lung Cancer.

[41] Kong F.-M., Ritter T., Quint D.J., Senan S., Gaspar L.E., Komaki R.U. (2011). Consideration of dose limits for organs at risk of thoracic radiotherapy: atlas for lung, proximal bronchial tree, esophagus, spinal cord, ribs, and brachial plexus. Int J Radiat Oncol Biol Phys.

[42] Astone P, Pizzella G. Upper Limits in the Case That Zero Events are Observed: An Intuitive Solution to the Background Dependence Puzzle. Published online February 10, 2000. doi:10.48550/arXiv.hep-ex/0002028.

[43] Andratschke N., Willmann J., Appelt A.L., Alyamani N., Balermpas P., Baumert B.G. (2022). European Society for Radiotherapy and Oncology and European Organisation for Research and Treatment of Cancer consensus on re-irradiation: definition, reporting, and clinical decision making. Lancet Oncol.

[44] Wang C., Rimner A., Gelblum D.Y., Dick-Godfrey R., McKnight D., Torres D. (2020). Analysis of pneumonitis and esophageal injury after stereotactic body radiation therapy for ultra-central lung tumors. Lung Cancer Amst Neth.

[45] Arcidiacono F., Anselmo P., Casale M., Zannori C., Ragusa M., Mancioli F. (2023). STereotactic Ablative RadioTherapy in NEWly diagnosed and recurrent locally advanced non-small cell lung cancer patients unfit for concurrEnt RAdio-chemotherapy: early analysis of the START-NEW-ERA non-randomised phase II trial. Int J Radiat Oncol Biol Phys.

[46] Giuliani M., Mathew A.S., Bahig H., Bratman S.V., Filion E., Glick D. (2018). SUNSET: Stereotactic Radiation for Ultracentral Non-Small-Cell Lung Cancer-A Safety and Efficacy Trial. Clin Lung Cancer.

