# Supplementary material for: Dosimetric Analysis of Proximal Bronchial Tree Subsegments to Assess The Risk of Severe Toxicity After Stereotactic Body Radiation Therapy of Ultra-central Lung Tumors
Source: Clin Transl Radiat Oncol. 2023 Dec 7;45:100707. doi: 10.1016/j.ctro.2023.100707 (PMC10731610; doi:10.1016/j.ctro.2023.100707)
Supplement: Supplementary data 1 [file mmc1.docx]

**Supplementary tables**

**Table 5: Univariate and multivariate Cox regression analysis of parameters associated with overall survival and local control**

| Endpoint | **Overall survival HR (95% CI, P value)** | | **Local control HR (95% CI, P value)** | |
| --- | --- | --- | --- | --- |
| *Parameter* | *Univariate analysis* | *Multivariate analysis* | *Univariate analysis* | *Multivariate analysis* |
| GTV size | **1.02 (1.0-1.04, p=0.009)** | 0.96 (0.90-1.02, p=0.2) | 1.02 (0.99-1.04, p=0.08) | 0.98 (0.89-1.07, p=0.72) |
| PTV size | **1.01 (1.0-1.02, p=0.003)** | **1.04 (1.0-1.07, p=0.037)** | 1.01 (1.0-1.02, p=0.06) | 0.99 (0.94-1.05, p=0.84) |
| Tumor size | **1.42 (1.17-1.73, p=0.0005)** | **1.49 (1.19-1.87, p=0.0005)** | **1.38 (1.05-1.83, P=0.0023)** | 1.95  (0.83-4.6, p=0.12) |
| Age | **1.05 (1.01-1.1, p=0.013)** | **1.07 (1.02-1.12, p=0.0035)** | **-** | - |
| Gender (female) | 0.68 (0.28-1.67, p=0.41) | 0.61 (0.24-1.55, p=0.3) | **-** | - |
| Total dose delivered to tumor (EQD2_10) | 0.98 (0.95-1.01, p=0.41) | 0.99 (0.95-1.03, p=0.53) | 1.01 (0.96-1.05, p=0.84) | 1.03 (0.98-1.07, p=0.26) |
| EQD2_10 to PTV below 54.37 Gy  No  Yes | -  - | -  - | *Reference*  1.2 (0.4-3.3, p=0.77) | *Reference*  1.2 (0.3-5.8, p=0.8) |
| D0.1cc of PTV (EQD2_10) | - | - | 1 (0.97-1, p=0.9) | 1.0 (0.97-1, p=0.9) |
| D0.1cc of PTV below 86.5 Gy (EQD2_10)  No  Yes | -  - | -  - | *Reference*  1.5 (0.5-4.1, p=0.5) | *Reference*  4.5 (0.6-29.0, p=0.2) |
| V100% of PTV (%) | - | - | 1 (0.93-1.1, 0.68) | 1 (0.9-1.2, p=0.6) |
| V100% of PTV (%) below 95%  No  Yes | -  - | -  - | *Reference*  0.5 (0.1-3.6, p=0.5) | *Reference*  0.5 (0.1-3.9, p=0.5) |
| Previous thoracic radiotherapy | 1.0 (0.43-2.34, p=0.99) | 2.28 (0.71-6.93, p=0.26) | 1.81 (0.61-5.3, p=0.28) | 2.38 (0.71-7.93, p=0.16) |
| D0.2cc of PBT (EQD2) | 1.0 (0.99-1.01, p=0.71) | 1.0 (0.99-1.02, p=0.41) | - | - |

*Specification of model: GTV- and PTV size, tumor size, age, total dose delivered to tumor, D0.2cc to PBT were coded as continuous variables. Gender and previous thoracic radiotherapy were coded as categorical variables.*

**Table 6: Internal dose constraints**

| **SBRT 5 fractions** | | | | | **SBRT 8 fractions** | | | | |
| --- | --- | --- | --- | --- | --- | --- | --- | --- | --- |
| Structure | Priority | Parameter |  | SBRT 5 fractions | Structure | Priority | Parameter |  | SBRT 8 fractions |
| PTV | 2 | V100 [% of volume] |  | ≥ 95.00 | PTV | 2 | V100 [% of volume] | | ≥ 95.00 |
| PTV | 2 | CI |  | ≤ 1.20 | PTV | 2 | CI |  | ≤ 1.20 |
| PTV | 2 | CI |  | > 1.00 | PTV | 2 | CI |  | > 1.00 |
| ITV | 2 | V110 [% of volume] |  | x | ITV | 2 | V110 [% of volume] | | x |
| ITV | 2 | V135 [% of volume] |  | ≥ 95.00 | ITV | 2 | V135 [% of volume] | | ≥ 95.00 |
| ITV | 2 | D0.1cc [% of dose] |  | ≤ 156.00 | ITV | 2 | D0.1cc [% of dose] | | ≤ 156.00 |
| ITV | 2 | D0.1cc [% of dose] |  | ≥ 152.00 | ITV | 2 | D0.1cc [% of dose] | | ≥ 152.00 |
|  |  |  |  | **Total dose (Gy), EQD2, alpha/beta=3** |  |  |  |  | **Total dose (Gy), EQD2, alpha/beta=3** |
| Lung | 3 | Dmean [Gy] | ≤ | 10 | Lung | 3 | Dmean [Gy] | ≤ | 18 |
| BrachialPlexus_L | 1 | D0.1cc [Gy] | ≤ | 40 | BrachialPlexus_L | 1 | D0.1cc [Gy] | ≤ | 54 |
| BrachialPlexus_R | 1 | D0.1cc [Gy] | ≤ | 40 | BrachialPlexus_R | 1 | D0.1cc [Gy] | ≤ | 54 |
| BronchialTree | 1 | D0.1cc [Gy] | ≤ | 80.56 | BronchialTree | 1 | D0.1cc [Gy] | ≤ | 112.24 |
| ThoracicWall | 3 | D30cc [Gy] | ≤ | 37.44 | ThoracicWall | 3 | D30cc [Gy] | ≤ | 42.64 |
| ThoracicWall | 3 | D5cc [Gy] | ≤ | 108 | ThoracicWall | 3 | D5cc [Gy] | ≤ | 149.04 |
| Esophagus | 3 | D1cc [Gy] | ≤ | 42.64 | Esophagus | 3 | D1cc [Gy] | ≤ | 54 |
| Esophagus | 1 | D0.1cc [Gy] | ≤ | 54 | Esophagus | 1 | D0.1cc [Gy] | ≤ | 70 |
| Heart | 3 | D15cc [Gy] | ≤ | 54 | Heart | 3 | D15cc [Gy] | ≤ | 73.44 |
| Heart | 3 | D0.1cc [Gy] | ≤ | 73.44 | Heart | 3 | D0.1cc [Gy] | ≤ | 88 |
| Trachea | 3 | D0.1cc [Gy] | ≤ | 80.56 | Trachea | 3 | D0.1cc [Gy] | ≤ | 112.24 |
| Kidney | 3 | Dmean [Gy] | ≤ | 6.16 | Kidney | 3 | Dmean [Gy] | ≤ | 7.36 |
| Kidney | 3 | D75% [Gy] | ≤ | 18 | Kidney | 3 | D75% [Gy] | ≤ | 18 |
| Stomach | 1 | D1cc [Gy] | ≤ | 23.76 | Stomach | 1 | D1cc [Gy] | ≤ | 30.24 |
| Duodenum | 1 | D1cc [Gy] | ≤ | 23.76 | Duodenum | 1 | D1cc [Gy] | ≤ | 30.24 |
| Bowel | 1 | D1cc [Gy] | ≤ | 23.76 | Bowel | 1 | D1cc [Gy] | ≤ | 30.24 |
| Stomach | 1 | D0.1cc [Gy] | ≤ | 28 | Stomach | 1 | D0.1cc [Gy] | ≤ | 37.44 |
| Duodenum | 1 | D0.1cc [Gy] | ≤ | 28 | Duodenum | 1 | D0.1cc [Gy] | ≤ | 37.44 |
| Bowel | 1 | D0.1cc [Gy] | ≤ | 28 | Bowel | 1 | D0.1cc [Gy] | ≤ | 37.44 |

**Table 7: Detailed individual-level description of radiotherapy including dosimetric specification of PTV, number of fractionation, single dose, total dose, dose in EQD2 and radiation timing**

| ID | Radiation timing | Fractions (n) | Single dose (Gy) | Total dose (physical) in Gy | Prescription isode in % | EQD2 (alpha/beta=10) in Gy | V100% of PTV (%) | D0.1cc of PTV (EQD2, alpha/beta=10) in Gy | DMAX (point dose) of PTV (EQD2, alpha/beta=10) in Gy |
| --- | --- | --- | --- | --- | --- | --- | --- | --- | --- |
| 1 | Daily | 12 | 5 | 60.0 | 80 | 75.0 | 98.8 | 104.1 | 108.1 |
| 2 | Daily | 10 | 5 | 50.0 | 80 | 62.5 | 95.0 | 87.8 | 89.9 |
| 3 | Daily | 10 | 5 | 50.0 | 80 | 62.5 | 98.5 | 87.3 | 88.7 |
| 4 | Daily | 10 | 5 | 50.0 | 80 | 62.5 | 99.0 | 86.5 | 88.4 |
| 5 | Daily | 10 | 5 | 50.0 | 80 | 62.5 | 99.0 | 86.2 | 87.6 |
| 6 | Daily | 10 | 5 | 50.0 | 80 | 62.5 | 96.0 | 84.3 | 85.7 |
| 7 | Daily | 10 | 5 | 50.0 | 80 | 62.5 | 96.0 | 83.1 | 84.3 |
| 8 | Daily | 10 | 5 | 50.0 | 80 | 62.5 | 98.0 | 82.4 | 84.0 |
| 9 | Daily | 10 | 5.5 | 55.0 | 80 | 71.0 | 97.9 | 74.5 | 81.6 |
| 10 | Daily | 10 | 4.5 | 45.0 | 80 | 54.4 | 98.8 | 73.2 | 75.2 |
| 11 | Daily | 10 | 4.5 | 45.0 | 80 | 54.4 | 93.2 | 73.1 | 75.9 |
| 12 | Daily | 10 | 4.5 | 45.0 | 80 | 54.4 | 95.8 | 73.1 | 74.5 |
| 13 | Daily | 10 | 4.5 | 45.0 | 80 | 54.4 | 97.5 | 72.0 | 72.9 |
| 14 | Daily | 10 | 4.5 | 45.0 | 80 | 54.4 | 98.0 | 71.8 | 72.8 |
| 15 | Daily | 10 | 4 | 40.0 | 80 | 46.7 | 96.5 | 65.5 | 66.1 |
| 16 | Daily | 10 | 4 | 40.0 | 80 | 46.7 | 98.5 | 63.4 | 64.1 |
| 17 | Daily | 10 | 4 | 40.0 | 80 | 46.7 | 98.0 | 63.3 | 64.9 |
| 18 | Daily | 10 | 4 | 40.0 | 80 | 46.7 | 96.5 | 62.7 | 63.3 |
| 19 | Daily | 10 | 4 | 40.0 | 80 | 46.7 | 96.0 | 62.3 | 62.7 |
| 20 | Daily | 10 | 4.6 | 46.0 | 80 | 56.0 | 94.0 | 61.3 | 62.2 |
| 21 | Daily | 10 | 4.5 | 45.0 | 80 | 54.4 | 95.6 | 53.1 | 54.0 |
| 22 | Daily | 10 | 4.25 | 42.5 | 80 | 50.5 | 74.2 | 53.0 | 54.2 |
| 23 | Daily | 10 | 3 | 30.0 | 80 | 32.5 | 99.0 | 43.1 | 45.0 |
| 24 | Daily | 9 | 5 | 45.0 | 65 | 56.3 | 95.0 | 60.6 | 63.0 |
| 25 | Daily | 8 | 7.5 | 60.0 | 65 | 87.5 | 95.0 | 120.6 | 128.2 |
| 26 | Daily | 8 | 6 | 48.0 | 65 | 64.0 | 96.0 | 120.0 | 124.5 |
| 27 | Daily | 8 | 5 | 40.0 | 65 | 50.0 | 91.6 | 119.3 | 121.8 |
| 28 | Daily | 8 | 6 | 48.0 | 65 | 64.0 | 99.3 | 119.0 | 120.7 |
| 29 | Daily | 8 | 6 | 48.0 | 65 | 64.0 | 87.2 | 118.8 | 132.4 |
| 30 | Daily | 8 | 6 | 48.0 | 65 | 64.0 | 96.0 | 118.2 | 123.2 |
| 31 | Daily | 8 | 6 | 48.0 | 65 | 64.0 | 95.0 | 117.5 | 119.8 |
| 32 | Daily | 8 | 6 | 48.0 | 65 | 64.0 | 96.3 | 117.4 | 119.3 |
| 33 | Daily | 8 | 6 | 48.0 | 65 | 64.0 | 98.5 | 117.1 | 120.2 |
| 34 | Daily | 8 | 6 | 48.0 | 65 | 64.0 | 99.4 | 116.6 | 119.4 |
| 35 | Daily | 8 | 6 | 48.0 | 65 | 64.0 | 96.0 | 115.3 | 119.4 |
| 36 | Daily | 8 | 6 | 48.0 | 65 | 64.0 | 97.0 | 114.4 | 119.3 |
| 37 | Daily | 8 | 7.5 | 60.0 | 65 | 87.5 | 95.0 | 113.1 | 117.6 |
| 38 | Daily | 8 | 7.5 | 60.0 | 65 | 87.5 | 95.0 | 112.9 | 120.1 |
| 39 | Daily | 8 | 5.5 | 44.0 | 65 | 56.8 | 95.0 | 103.5 | 106.0 |
| 40 | Daily | 8 | 7.5 | 60.0 | 65 | 87.5 | 95.0 | 92.9 | 93.4 |
| 41 | Daily | 8 | 5 | 40.0 | 65 | 50.0 | 99.0 | 91.9 | 93.5 |
| 42 | Daily | 8 | 5 | 40.0 | 65 | 50.0 | 95.5 | 91.5 | 94.9 |
| 43 | Daily | 8 | 5 | 40.0 | 65 | 50.0 | 99.4 | 91.2 | 93.0 |
| 44 | Daily | 8 | 5 | 40.0 | 65 | 50.0 | 98.5 | 90.8 | 92.2 |
| 45 | Daily | 8 | 5 | 40.0 | 65 | 50.0 | 99.0 | 90.3 | 95.5 |
| 46 | Daily | 8 | 6 | 48.0 | 65 | 64.0 | 95.8 | 90.2 | 91.8 |
| 47 | Daily | 8 | 5 | 40.0 | 65 | 50.0 | 99.0 | 90.0 | 91.4 |
| 48 | Daily | 8 | 5 | 40.0 | 65 | 50.0 | 96.7 | 89.9 | 91.6 |
| 49 | Daily | 8 | 5 | 40.0 | 65 | 50.0 | 99.2 | 77.5 | 78.3 |
| 50 | Daily | 8 | 5 | 40.0 | 65 | 50.0 | 95.0 | 70.7 | 74.1 |
| 51 | Daily | 8 | 5 | 40.0 | 65 | 50.0 | 99.0 | 69.8 | 72.5 |
| 52 | Daily | 8 | 5 | 40.0 | 65 | 50.0 | 97.4 | 68.6 | 69.7 |
| 53 | Daily | 8 | 5 | 40.0 | 65 | 50.0 | 96.0 | 66.6 | 70.6 |
| 54 | Daily | 8 | 4 | 32.0 | 65 | 37.3 | 95.7 | 49.5 | 49.9 |
| 55 | Daily | 7 | 6 | 42.0 | 65 | 56.0 | 91.8 | 101.6 | 104.8 |
| 56 | Daily | 6 | 5 | 30.0 | 65 | 37.5 | 95.0 | 63.6 | 67.4 |
| 57 | Daily | 5 | 6 | 30.0 | 65 | 40.0 | 95.0 | 72.0 | 75.1 |

**Table 8:** *Detailed description of doses in EQD2_3 to thoracic OARs*

| **Structure** | **Dose measured (EQD2, α/β = 3 Gy)** | **Median in Gy (range)** |
| --- | --- | --- |
| Trachea | D_1cc_  D_0.5cc_  D_0.01cc_  D_max_ | 5.7 (0.3-71.6)  7.4 (0.3-81.0)  15.6 (0.3-112.2)  16.9 (0.3-116.6) |
| Proximal bronchial tree | D_1cc_  D_0.5cc_  D_0.01cc_  D_max_ | 59.3 (39.2-125.3)  69.2 (43.5-131.8)  98.4 (45.1-170.7)  103.7 (46.2-174.0) |
| **PBT sub-structures overlapping with PTV** |  |  |
| - ***Distal 2 cm of trachea*** | D_1cc_  D_0.5cc_  D_0.01cc_  D_max_ | 4.8 (0.2-62.2)  6.7 (0.2-69.5)  14.3 (0.2-170.7)  32.2 (0.2-174.0) |
| - ***Right main bronchus*** | D_1cc_  D_0.5cc_  D_0.01cc_  D_max_ | 8.2 (0.1-93.4)  10.8 (0.1-104.5)  16.0 (0.1-169.0)  20.7 (0.1-172.3) |
| - ***Proximal left main bronchus*** | D_1cc_  D_0.5cc_  D_0.01cc_  D_max_ | 7.8 (0.1-123.0)  13.4 (0.1-131.4)  22.1 (0.1-169.6)  26.9 (0.1-171.5) |
| - ***Right intermediate bronchus*** | D_1cc_  D_0.5cc_  D_0.01cc_  D_max_ | 5.1 (0.04-106.0)  6.3 (0.05-125.5)  8.5 (0.06-146.7)  8.8 (0.06-156.7) |
| - ***Distal left main bronchus*** | D_1cc_  D_0.5cc_  D_0.01cc_  D_max_ | 4.5 (0.02-85.5)  5.0 (0.04-94.3)  6.3 (0.08-151.7)  6.6 (0.08-163.3) |
| - ***Right lower lobar bronchus*** | D_1cc_  D_0.5cc_  D_0.01cc_  D_max_ | 0.3 (0.04-83.6)  0.9 (0.04-100.8)  9.9 (0.04-141.5)  11.0 (0.04-160.0) |
| - ***Left lower lobar bronchus*** | D_1cc_  D_0.5cc_  D_0.01cc_  D_max_ | 0.5 (0.04-4.1)  0.5 (0.04-9.0)  4.9 (0.04-134.5)  5.1 (0.04-144.6) |
