# Supplementary material for: Dosimetric Analysis of Proximal Bronchial Tree Subsegments to Assess The Risk of Severe Toxicity After Stereotactic Body Radiation Therapy of Ultra-central Lung Tumors
Source: Clin Transl Radiat Oncol. 2023 Dec 7;45:100707. doi: 10.1016/j.ctro.2023.100707 (PMC10731610; doi:10.1016/j.ctro.2023.100707)

**Supplementary material**

*Statistical analysis and detailed dose response model description*

The dose response model for grade ≥3 bronchial stenosis, hemorrhage or fistula was built using Bayesan inference. Previous meta-analysis showed an expected complication probability (CP) of 10% at 88 Gy.[^39^](https://www.zotero.org/google-docs/?kwt1wC) A logistic prior distribution with expected value CP=10% and 95% confidence interval (CI) up to CP=20% was adopted for doses between 0-88 Gy. For doses >88 Gy the prior was uniform for CP between 0-100%, therefore without constraining CP to any upper limit. The likelihood was computed based on a binomial distribution taking into account the number of observed patients without toxicity. The expected value for CP and the CI (0-95%) were extracted from the posterior distribution. The evaluation of CI up to 95% was chosen to allow a direct comparison of this study with the results reported by the *Nordic HILUS trial*[^40^](https://www.zotero.org/google-docs/?jYfIC4). Not only the number of patients without toxicity was taken as an input parameter, but also the follow-up time, which was previously reported as critical to determine the maximum tolerated dose.[^42^](https://www.zotero.org/google-docs/?NXDvOx) The overall dose response model was built using the minimum follow-up time (0.63 years) while the confidence interval dependence on the follow-up time was analyzed for a specific dose level, namely 100 Gy. The NTCP and time to toxicity of this study were compared to the *Nordic HILUS Trial*[^40^](https://www.zotero.org/google-docs/?19DBwx). The former was performed taking into account the full cohorts. The latter was performed comparing the follow-up of the patients in the Group A (target lesion within 10 mm of PBT, trachea or main bronchi) of the *Nordic HILUS trial*[^40^](https://www.zotero.org/google-docs/?8C6Kee) with the patients receiving 100 Gy or more in the current study.

**Figure 3: Cumulative incidence of events of non-metastatic NSCLC vs OMD and recurrent NSCLC**

**p=0.017 (Gray`s test), the CMPRSK model captured the first event only, therefore all death events were without evidence of disease.*


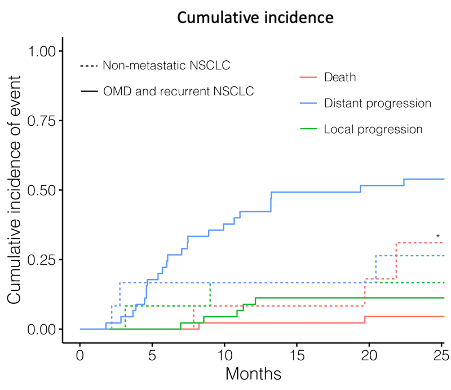

Supplement: Supplementary data 2 [file mmc2.docx]
